# Decatecholaminization and Calcium Sensitizers in Critically Ill Patients

**DOI:** 10.5812/cardiovascmed.16714

**Published:** 2014-02-24

**Authors:** Samad E J Golzari, Ata Mahmoodpoor

**Affiliations:** 1 Neuroscience Research Center, Institute of Neuropharmacology, Kerman University of Medical Sciences, Kerman, IR Iran; 2 Cardiovascular Research Center, Tabriz University of Medical Sciences, Tabriz, IR Iran

**Keywords:** Sepsis, Adrenergic beta-Antagonists

Sepsis-associated cardiac complications contribute to a major increase in mortality and morbidity of critically ill patients (1). Sepsis is often characterized by increased catecholamine levels and associated with increased cardiac contractility and heart rate. Throughout the disease procedure, mitochondrial dysfunction leads to supply/demand imbalance increasing the risk of cardiac myocytes death. However, reducing cell-specific functions enables cells to balance energy production and demand. Through this down-regulation, cardiomyocytes survive in a hibernation-like state and when the septic insult is overcome and cellular energy supply reestablished, the contraction is restarted (2). Decatecholaminization, the reduction of endogenous and exogenous adrenergic stimulation, has been accepted to be important in the management of critically ill patients particularly in the transition between acute and chronic critical illness; beta-blockers are considered as a choice option in this respect. Therefore, beta blockers should be titrated in septic patients and close hemodynamic monitoring is warranted for early detection of potential negative effects (3). For instance, administration of esmolol has been shown to be associated with reduction in heart rate without increasing the adverse events (4).

Another approach of cardioprotection in septic patients is reducing afterload for an already dysfunctional left ventricle, as high afterload could increase cardiomyocytes workload and impair the supply/demand balance. Levosimendan, a calcium sensitizer, might be the drug of choice for inotropic effects required in patients with beta-blockers, as beta-agonists reduce the response and impair the supply/demand balance due to tachycardia. Levosimendan is of anti-ischemic, anti-inflammatory and anti-apoptotic properties; consequently, modulating crucial pathways in the pathophysiology of septic shock (5, 6). Due to these advantageous impacts, levosimendan positively reinforces myocardial performance and regional hemodynamics improving the microcirculatory perfusion (7, 8). In a recent study, it was demonstrated that if mixed venous oxygen saturation decreased to less than 65% despite appropriate arterial oxygenation (≥ 95%) and hemoglobin concentrations of 8 g/dL or higher, arterial lactate concentrations increased, or both, levosimendan administration improved systemic oxygen delivery at a dose of 0.2 μg/kg/min (without a loading bolus dose) for 24 hours. Overall, critically ill patients could benefit from beta-blocker therapy in combination with calcium sensitizers if all precautionary measures are considered.
